# Supplementary material for: Adaptive and non-adaptive divergence in a common landscape
Source: Nat Commun. 2017 Aug 16;8:267. doi: 10.1038/s41467-017-00256-6 (PMC5559485; doi:10.1038/s41467-017-00256-6)
Supplement: Supplementary file 1 — Supplementary Information [file 41467_2017_256_MOESM1_ESM.pdf]

File name: Supplementary Information
Description: Supplementary Figures, Supplementary Tables, Supplementary Methods and Supplementary References

File name: Peer Review File
Description:

SUPPLEMENTARY FIGURES

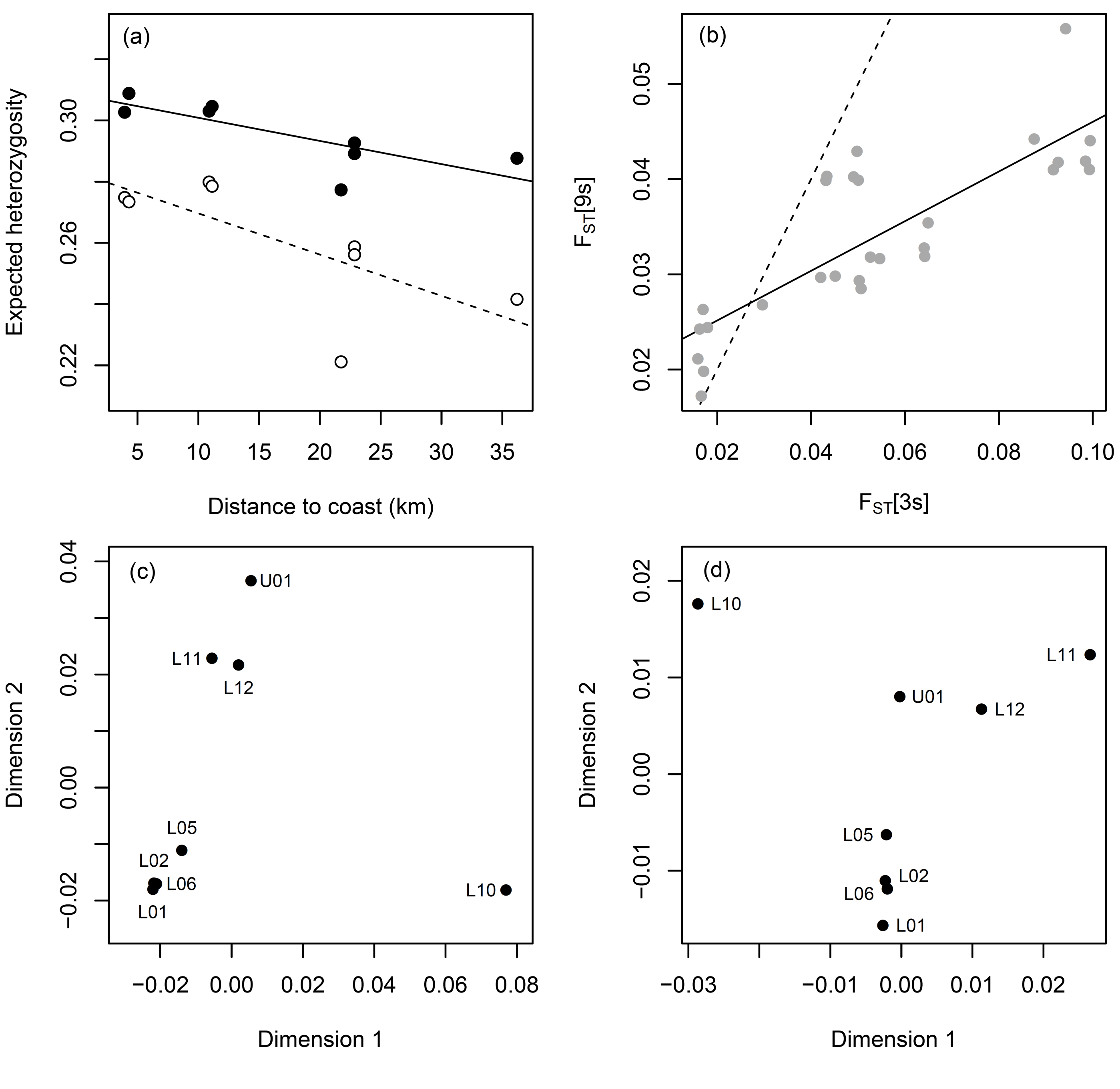

Supplementary Figure 1 │ Neutral population genetic structure among populations of the three-spined (3s) and nine-spined (9s) stickleback based on 12,684 and 10,0068 neutral SNPs, respectively. (a) Genetic diversity, quantified as expected heterozygosity, declines with distance to the coast in both species (3s: white dots and dashed regression line; slope = -0.0013; P = 0.0432; 9s: black dots and full regression line; slope = -0.0008; P = 0.0219). (b) Pairwise F_{ST} values among populations of the three-spined stickleback correlate with pairwise F_{ST} values among the corresponding populations of the nine-spined stickleback (Mantel test; R = 0.82; P = 0.0017), even though overall genetic differentiation among nine-spined stickleback populations is lower. The full line and the dashed line represent the regression line and the 1:1 line, respectively. (c) Classical multidimensional scaling (CMDS) of pairwise F_{ST} in the three-spined stickleback. (d) CMDS of pairwise F_{ST} in the nine-spined stickleback.

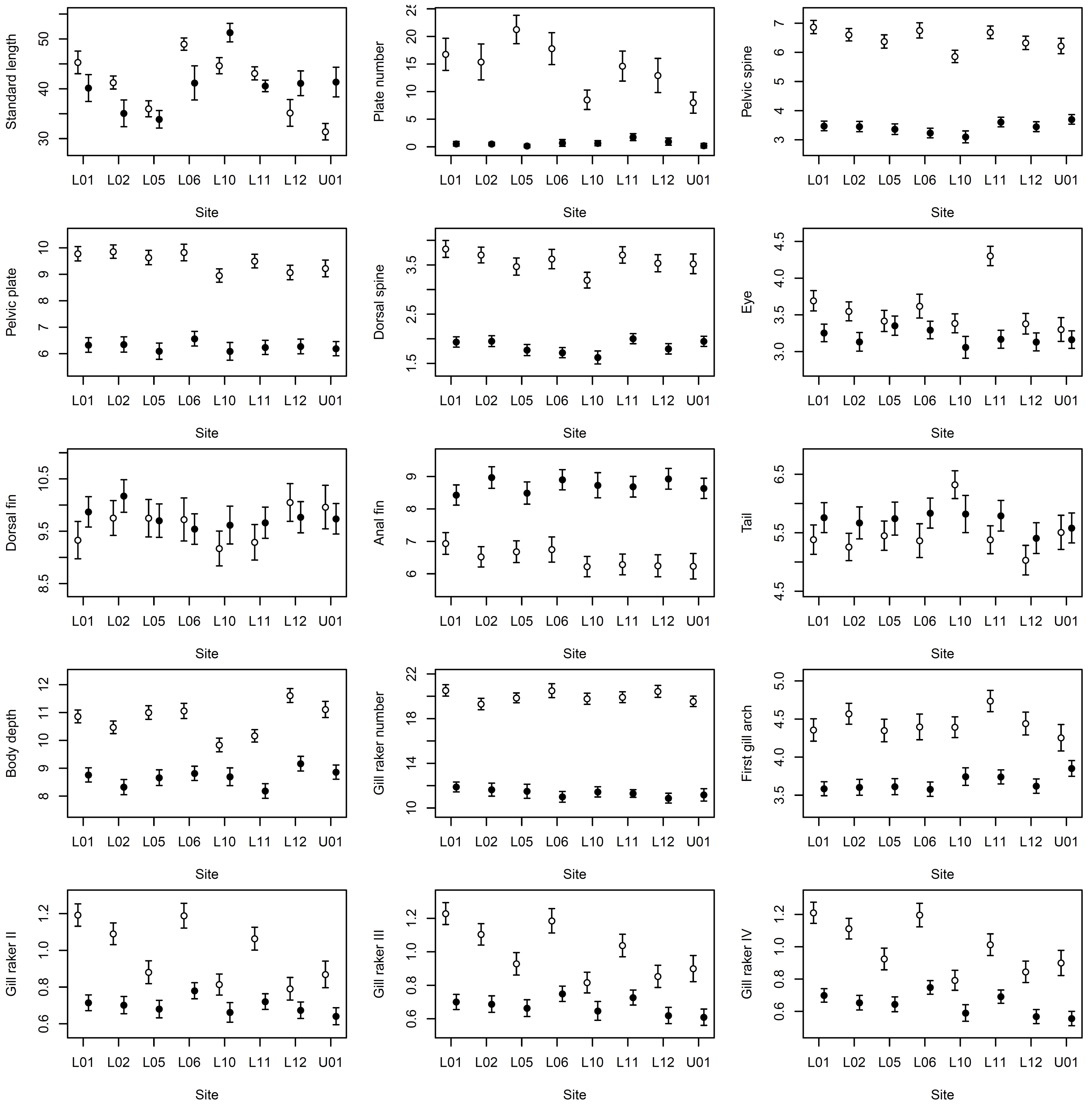

Supplementary Figure 2 | Averages for meristic traits and predicted values for metric traits in populations of the three-spined (white dots) and nine-spined stickleback (black dots) at eight sites in the Belgian-Dutch lowlands. Sites are ordered with respect to distance to the coast, and include four brackish sites (L01-L06) and four freshwater sites (L10-U01). Error bars represent 95% confidence intervals. Metric traits are displayed in mm and are predicted for a standard length or gill arch length (in case of gill raker II to IV) set at the overall mean of each species. In the three-spined stickleback, freshwater populations had fewer lateral plates (χ^2 = 10.9; df = 1; P = 0.0010), and shorter pelvic plates (χ^2 = 18.1; df = 1; P < 0.0001), anal fins (χ^2 = 15.7; df = 1; P < 0.0001), and gill rakers (e.g. gill raker IV: χ^2 = 7.6; df = 1; P = 0.0059) than the brackish water populations. In the nine-spined stickleback, freshwater populations had longer gill arches (χ^2 = 7.7; df = 1; P = 0.0056), shorter gill rakers (gill raker IV: χ^2 = 4.5; df = 1; P = 0.0346), and smaller eyes (χ^2 = 4.4; df = 1; P = 0.0365) than the brackish water populations.

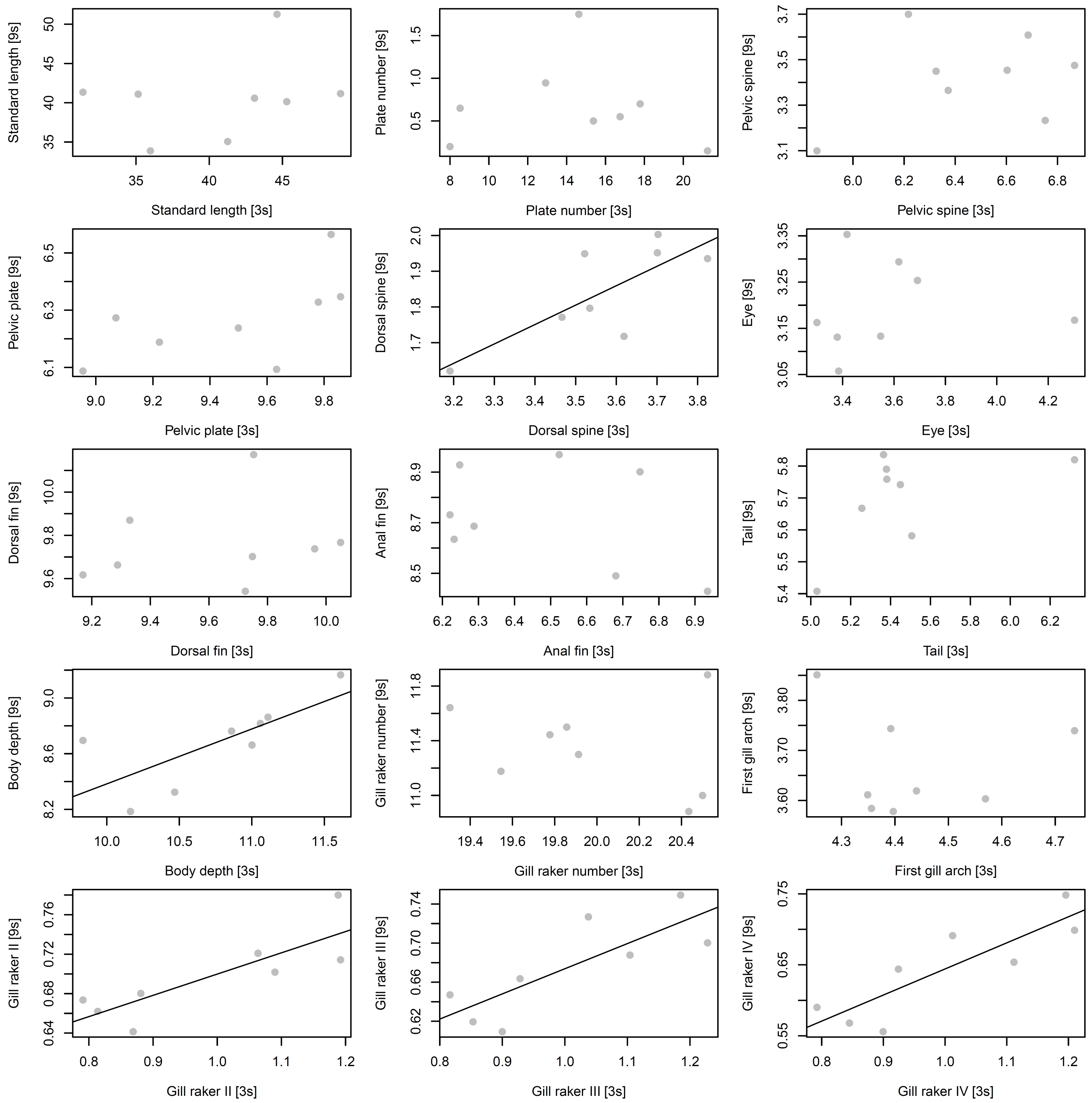

Supplementary Figure 3 | Regression of mean meristic traits or predicted metric traits between coexisting populations of the three-spined (3s) and nine-spined (9s) stickleback. Metric traits are displayed in mm and are predicted for a standard length or gill arch length (in case of gill raker II to IV) set at the overall mean of each species. Full lines (superimposed on significant relationships) represent least-square linear regression lines.

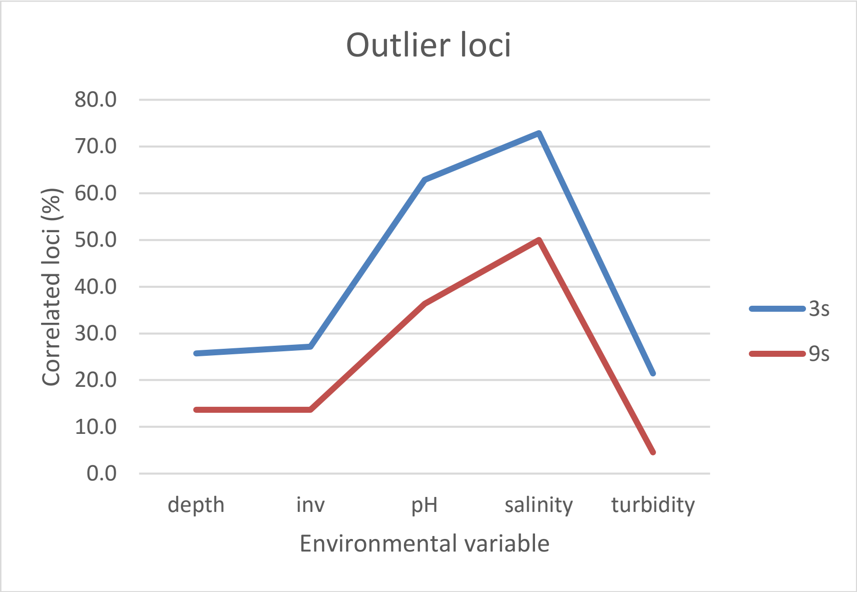

Supplementary Figure 4 | Adaptive significance of SNP loci in eight populations of the three-spined stickleback (3s) and eight populations of the nine-spined stickleback (9s). The adaptive significance of the SNPs is revealed by combining the results of BAYESCAN (identifying outlier loci) and latent factor mixed models (LFMM; identifying loci correlated with environmental variables). Environmental variables and outlier loci were more often correlated in the three-spined stickleback than in the nine-spined stickleback. Salinity and pH correlated with the largest percentage of outlier loci in both species.

SUPPLEMENTARY TABLES

Supplementary Table 1 | Characteristics of the eight sites in the Belgian-Dutch lowlands that harboured populations of both three-spined (3s) and nine-spined (9s) stickleback, together with information on densities, effective population size and sample sizes of the two species. DTC: distance to the coast. Inv: density of macro-invertebrate predators expressed as number of individuals per meter caught along the bank. Dens3s and Dens9s: density of the three-spined and nine-spined stickleback expressed as number of individuals per meter caught along the bank. The values for these biotic and abiotic variables represent the mean (± standard deviation) across four seasons (spring 2008, fall 2008, spring 2009 and summer 2009). N_e: effective population size (two values could not be estimated). N_{3s}: sample size for the three-spined stickleback; N_{9s}: sample size for the nine-spined stickleback. Values before and after the dash represent sample size for morphology (excluding individuals with missing data) and genomics (excluding individuals with low read number), respectively.

Site	Habitat	DTC (km)	Salinity (psu)	Depth (cm)	pH	Turbidity	Inv (ind m^{-1})	Dens3s (ind m^{-1})	Dens9s (ind m^{-1})	N_e[3s] (95 % CI)	N_e[9s] (95 % CI)	N_{3s}	N_{9s}
L01	creek	3.94	2.04 ± 0.61	33.06 ± 3.94	7.89 ± 0.33	-22.25 ± 6.70	0.04 ± 0.05	0.15 ± 0.13	0.52 ± 0.87	167 (165-169)	684 (643-732)	24/21	20/22
L02	creek	4.30	1.83 ± 0.88	33.19 ± 17.47	7.78 ± 0.27	-21.25 ± 4.57	0.01 ± 0.01	0.14 ± 0.07	0.30 ± 0.23	131 (130-132)	957 (888-1037)	24/24	20/23
L05	creek	10.90	1.04 ± 0.27	49.69 ± 9.06	7.69 ± 0.22	-25.46 ± 4.80	0.00 ± 0.00	0.23 ± 0.23	0.17 ± 0.07	150 (149-152)	175 (170-180)	24/23	20/18
L06	creek	11.14	2.35 ± 0.76	73.54 ± 20.49	7.69 ± 0.19	-20.00 ± 10.30	0.00 ± 0.00	0.06 ± 0.04	0.08 ± 0.09	143 (142-145)	218 (214-222)	23/23	20/23
L10	pond	21.75	0.32 ± 0.05	63.60 ± 2.80	7.45 ± 0.31	-19.67 ± 2.89	0.01 ± 0.01	0.34 ± 0.31	0.19 ± 0.14	82 (81-83)	n.a.	27/23	20/22
L11	pond	22.84	0.18 ± 0.03	71.65 ± 9.29	7.50 ± 0.28	-17.00 ± 3.56	0.16 ± 0.14	1.05 ± 0.81	0.55 ± 0.27	60.9 (60.6-61.2)	156 (154-159)	24/24	20/23
L12	stream	22.84	0.32 ± 0.05	40.25 ± 9.28	7.17 ± 0.37	-28.50 ± 5.07	0.01 ± 0.01	0.72 ± 0.22	0.35 ± 0.17	n.a.	767 (720-821)	25/13	19/22
U01	ditch	36.20	0.52 ± 0.14	56.63 ± 5.89	7.49 ± 0.25	-29.25 ± 11.53	0.03 ± 0.02	1.86 ± 2.26	0.60 ± 0.93	168 (166-170)	1822 (1574-2161)	25/24	20/22

Supplementary Table 2 | Single-species and two-species general linear models (ANOVA or ANCOVA) for 15 morphological traits (including body size) in eight populations of coexisting three-spined and nine-spined stickleback. Single-species models test for differences between sites in each species. Here, P-values in bold mark significant differences between sites, and R^2 values quantify the effect size of site. P_{ST} quantifies the overall phenotypic differentiation between sites (values in bold differ significantly from neutral F_{ST}). Two-species models test for site, species and the site by species interaction effect. Here, R^2[par] and R^2[npar] quantify the parallel (effect of site) and non-parallel (effect of site by species interaction) effect size, respectively. The R^2 ratio quantifies the relative importance of parallel vs. non-parallel effects, i.e., R^2 ratio = R^2[par]/R^2[npar]. ΔP_{ST} quantifies the difference in P_{ST} values between the three-spined stickleback and the nine-spined stickleback. The analyses correct for size differences between individuals by including standard length in models for PS, PP, DS, Eye, DF, AF, Tail, BD and GA, and by including gill arch length in models for LGR2, LGR3 and LGR4. Two-species models also include the species by size interaction effect.

			**Single-species models**								**Two-species models**			
			**Three-spined stickleback**				**Nine-spined stickleback**				**Three-spined vs. nine-spined stickleback**			
**Category**	**Trait**	**Code**	**F**	***P***	**R^2**	**P_{ST}**	**F**	***P***	**R^2**	**P_{ST}**	**R^2[par]**	**R^2[npar]**	**R^2 ratio**	**ΔP_{ST}**
Body size	Standard length	SL	$F_{7,188}$=47.65	**< 0.0001**	0.64	**0.49**	$F_{7,151}$=19.60	**< 0.0001**	0.48	**0.32**	0.37	0.20	1.87	0.17
Armour	Plate number	Plates	$F_{7,188}$=12.52	**< 0.0001**	0.32	**0.19**	$F_{7,151}$=4.64	**0.0001**	0.18	0.08	0.07	0.06	1.22	0.10
	Pelvic spine length	PS	$F_{7,187}$=9.04	**< 0.0001**	0.07	0.15	$F_{7,150}$=4.81	**0.0001**	0.10	0.09	0.02	0.01	2.99	0.05
	Pelvic plate length	PP	$F_{7,187}$=7.18	**< 0.0001**	0.03	0.12	$F_{7,147}$=1.23	0.2902	0.01	0.01	0.01	0.00	3.79	0.11
	Dorsal spine length	DS	$F_{7,185}$=5.90	**< 0.0001**	0.07	0.09	$F_{7,147}$=5.83	**< 0.0001**	0.12	0.12	0.02	0.00	8.60	-0.03
Body shape	Eye diameter	Eye	$F_{7,184}$=20.96	**< 0.0001**	0.22	**0.32**	$F_{7,149}$=2.14	**0.0430**	0.03	0.03	0.08	0.07	1.19	0.29
	Dorsal fin length	DF	$F_{7,187}$=2.74	**0.0099**	0.02	0.04	$F_{7,149}$=1.53	0.1613	0.01	0.01	0.01	0.01	1.24	0.02
	Anal fin length	AF	$F_{7,187}$=2.69	**0.0111**	0.03	0.04	$F_{7,149}$=1.62	0.1345	0.01	0.02	0.00	0.01	0.54	0.02
	Tail length	Tail	$F_{7,186}$=10.30	**< 0.0001**	0.15	**0.16**	$F_{7,149}$=1.19	0.3127	0.01	0.01	0.06	0.02	3.22	0.16
	Body depth	BD	$F_{7,153}$=20.77	**< 0.0001**	0.07	**0.37**	$F_{7,149}$=5.32	**< 0.0001**	0.03	0.10	0.03	0.01	5.05	0.27
Gills	Long gill raker number	NLGR	$F_{7,170}$=3.42	**0.0019**	0.12	0.05	$F_{7,126}$=2.12	**0.0465**	0.11	0.03	0.00	0.00	0.77	0.02
	First gill arch length	GA	$F_{7,185}$=4.39	**0.0002**	0.03	0.07	$F_{7,142}$=3.51	**0.0016**	0.02	0.07	0.01	0.01	1.27	0.00
	Length gill raker II	LGR2	$F_{7,187}$=23.36	**< 0.0001**	0.21	**0.38**	$F_{7,145}$=3.57	**0.0014**	0.06	0.07	0.10	0.04	2.50	0.31
	Length gill raker III	LGR3	$F_{7,187}$=19.23	**< 0.0001**	0.20	**0.31**	$F_{7,144}$=4.31	**0.0002**	0.08	0.09	0.09	0.03	3.02	0.23
	Length gill raker IV	LGR4	$F_{7,187}$=20.90	**< 0.0001**	0.21	**0.32**	$F_{7,145}$=9.33	**< 0.0001**	0.14	**0.19**	0.11	0.02	4.58	0.13

Supplementary Table 3 | Single-species and two-species MANCOVAs for three trait categories (armour, body shape or gill morphology) in coexisting three-spined (3s) and nine-spined (9s) stickleback from eight sites. Traits were grouped into categories according to Supplementary Table 2. Single-species models test for differences between sites in each species separately. Two-species models test for phenotypic parallelism (effect of site) and non-parallelism (effect of site by species). Standard length (SL) is included in the models to correct for size differences between individuals. Partial η^2 quantifies effect size. Significant P-values are in bold.

Category	Species	Effect	Df	Wilk's λ	F	*P*	partial η² (95 % CI)
Armour	3s and 9s	site	7	0.56	$F_{28,1176.8} = 7.26$	**< 0.0001**	0.134 (0.132 - 0.135)
		species	1	0.06	$F_{4,326} = 1323.96$	**< 0.0001**	0.942 (0.942 - 0.943)
		SL	1	0.29	$F_{4,326} = 196.19$	**< 0.0001**	0.707 (0.704 - 0.709)
		site by species	7	0.79	$F_{28,1176.8} = 2.78$	**< 0.0001**	0.056 (0.055 - 0.057)
		species by SL	1	0.86	$F_{4,326} = 13.37$	**< 0.0001**	0.141 (0.138 - 0.144)
		Residuals	329				
	3s	site	7	0.47	$F_{28,657.63} = 5.461$	**< 0.0001**	0.172 (0.169 - 0.175)
		SL	1	0.27	$F_{4,182} = 125.006$	**< 0.0001**	0.733 (0.729 - 0.737)
		Residuals	185				
	9s	site	7	0.56	$F_{28,509.8} = 3.192$	**< 0.0001**	0.136 (0.132 - 0.140)
		SL	1	0.30	$F_{4,141} = 80.549$	**< 0.0001**	0.696 (0.688 - 0.701)
		Residuals	144				
Body shape	3s and 9s	site	7	0.44	$F_{35,1243.4} = 7.67$	**< 0.0001**	0.152 (0.149 - 0.154)
		species	1	0.14	$F_{5,295} = 352.05$	**< 0.0001**	0.856 (0.859 - 0.859)
		SL	1	0.05	$F_{5,295} = 1239.48$	**< 0.0001**	0.955 (0.954 - 0.955)
		site by species	7	0.59	$F_{35,1243.4} = 4.72$	**< 0.0001**	0.1 (0.098 - 0.102)
		species by SL	1	0.86	$F_{5,295} = 9.41$	**< 0.0001**	0.138 (0.134 - 0.142)
		Residuals	299				
	3s	site	7	0.16	$F_{35,616.6} = 9.53$	**< 0.0001**	0.305 (0.301 - 0.310)
		SL	1	0.07	$F_{5,146} = 393.52$	**< 0.0001**	0.931 (0.929 - 0.933)
		Residuals	150				
	9s	site	7	0.59	$F_{35,612.39} = 2.36$	**< 0.0001**	0.101 (0.098 - 0.106)
		SL	1	0.03	$F_{5,145} = 976.27$	**< 0.0001**	0.971 (0.970 - 0.972)
		Residuals	149				
Gills	3s and 9s	site	7	0.48	$F_{35,1205.5} = 6.66$	**< 0.0001**	0.138 (0.136 - 0.140)
		species	1	0.05	$F_{5,286} = 1080.2$	**< 0.0001**	0.95 (0.949 - 0.950)
		SL	1	0.32	$F_{5,286} = 120.25$	**< 0.0001**	0.678 (0.673 - 0.682)
		site by species	7	0.66	$F_{35,1205.5} = 3.64$	**< 0.0001**	0.081 (0.080 - 0.083)
		species by SL	1	0.95	$F_{5,286} = 2.94$	**0.0131**	0.049 (0.046 - 0.051)
		Residuals	290				
	3s	site	7	0.31	$F_{35,696.52} = 6.368$	**< 0.0001**	0.208 (0.206 - 0.212)
		SL	1	0.36	$F_{5,165} = 57.437$	**< 0.0001**	0.635 (0.624 - 0.643)
		Residuals	169				
	9s	site	7	0.44	$F_{35,494.6} = 3.077$	**< 0.0001**	0.153 (0.149 - 0.157)
		SL	1	0.20	$F_{5,117} = 93.84$	**< 0.0001**	0.8 (0.794 - 0.806)
		Residuals	121				

Supplementary Table 4 | Number of SNPs, SNP-based neutral genetic differentiation (F_{ST}), and the percentage of outlier loci in eight populations of the three-spined (3s) and nine-spined (9s) stickleback. ODM: outlier detection method used to distinguish neutral from non-neutral (outlier) SNPs. Numbers separated by dashes refer to the number of neutral SNPs, outlier SNPs and all SNPs. Outlier SNPs only include loci showing higher genetic differentiation than expected, since such loci are potential targets of directional selection (loci with extremely low genetic differentiation were classified as neutral loci). In order to quantify the importance of outlier loci in the three-spined stickleback relative to the nine-spined stickleback (Outliers 3s vs. 9s), the percentage of outlier loci in the three-spined stickleback was divided by the percentage of outlier loci in the nine-spined stickleback.

ODM	Species	Number of SNPs	Neutral F_{ST} (± 95 % CI)	% outlier SNPs	Outliers 3s vs. 9s
LOSITAN	3s	12471/283/12754	0.058 (0.057-0.059)	2.22	10.57
	9s	10069/21/10090	0.033 (0.032-0.034)	0.21	
ARLEQUIN	3s	12688/66/12754	0.073 (0.072-0.074)	0.52	6.50
	9s	10082/8/10090	0.041 (0.040-0.041)	0.08	
BAYESCAN	3s	12684/70/12754	0.078 (0.076-0.080)	0.55	2.50
	9s	10068/22/10090	0.040 (0.039-0.041)	0.22	

Supplementary Table 5 | Gene ontology terms: shared and unique biological processes (BP) and molecular functions (MF) in the three-spined (3s) and the nine-spined (9s) stickleback. Numbers refer to the number of genes associated with specific BP and MF in each species.

Category	Term	3s	9s
Shared BP	metabolic process (GO:0008152)	17	1
	cellular process (GO:0009987)	16	2
	cellular component organization or biogenesis (GO:0071840)	8	2
	localization (GO:0051179)	5	2
Unique BP 3s	response to stimulus (GO:0050896)	3	-
	developmental process (GO:0032502)	3	-
	biological regulation (GO:0065007)	2	-
	multicellular organismal process (GO:0032501)	2	-
	biological adhesion (GO:0022610)	1	-
	locomotion (GO:0040011)	1	-
Unique BP 9s	-	-	-
Shared MF	catalytic activity (GO:0003824)	15	1
	binding (GO:0005488)	11	2
Unique MF 3s	transporter activity (GO:0005215)	4	-
	structural molecule activity (GO:0005198)	3	-
Unique MF 9s	-	-	-

Supplementary Table 6 | Partitioning of morphological and genomic variation in eight populations of the three-spined stickleback. The variation in morphology and genomics is partitioned in environmental (ENV) and spatial (SPACE) components. For morphology, analyses were conducted for all 14 traits combined as well as for each trait category separately (armour, body shape and gill morphology; see Supplementary Table 2). Here, the dependent variables are the individual size-corrected phenotypic traits (residuals of a linear regression of each trait on standard length or gill arch length). For genomics, analyses were conducted for the neutral loci as well as for the outlier loci. Here, the dependent variables are the first principal components (PC_1 to PC_n) of the neutral and outlier SNPs, where n is the number of principal components needed to include 50 % of the total allelic variation. Environmental dimensions include salinity, water depth, pH, turbidity and macroinvertebrate predators (see Supplementary Table 1). Spatial dimensions include X1 and X2, which represent the first and second dimension of a classical multidimensional scaling analysis on waterway distances. The unique and shared fractions explained by the respective variance components are represented by adjusted R^2 (R^2 adj), and by R^2 for each explanatory variable. Significant P-values are in bold. N: sample size. TV: total variation.

	**All traits**		**Armour**		**Body shape**		**Gill morphology**		**Neutral loci**		**Outlier loci**	
**Effect**	**R^2 adj**	***P***	**R^2 adj**	***P***	**R^2 adj**	***P***	**R^2 adj**	***P***	**R^2 adj**	***P***	**R^2 adj**	***P***
N	150		194		159		178		175		175	
TV	2086		772		790		885		171562		1602	
ENV	0.28	**< 0.0001**	0.15	**< 0.0001**	0.25	**< 0.0001**	0.27	**< 0.0001**	0.21	**< 0.0001**	0.74	**< 0.0001**
SPACE	0.15	**< 0.0001**	0.14	**< 0.0001**	0.12	**< 0.0001**	0.12	**< 0.0001**	0.14	**< 0.0001**	0.45	**< 0.0001**
ENV + SPACE	0.31	**< 0.0001**	0.21	**< 0.0001**	0.27	**< 0.0001**	0.28	**< 0.0001**	0.26	**< 0.0001**	0.82	**< 0.0001**
ENV|SPACE	0.17	**< 0.0001**	0.07	**< 0.0001**	0.15	**< 0.0001**	0.16	**< 0.0001**	0.13	**< 0.0001**	0.37	**< 0.0001**
SPACE|ENV	0.03	**< 0.0001**	0.05	**< 0.0001**	0.02	**0.0033**	0.02	**0.0070**	0.05	**< 0.0001**	0.08	**< 0.0001**
Residuals	0.69		0.79		0.73		0.72		0.74		0.18	
**Variable**	**R^2**	***P***	**R^2**	***P***	**R^2**	***P***	**R^2**	***P***	**R^2**	***P***	**R^2**	***P***
Salinity	0.14	**< 0.0001**	0.09	**< 0.0001**	0.05	**< 0.0001**	0.19	**< 0.0001**	0.10	**< 0.0001**	0.55	**< 0.0001**
Invertebrates	0.08	**< 0.0001**	0.05	**< 0.0001**	0.11	**< 0.0001**	0.06	**< 0.0001**	0.04	**< 0.0001**	0.06	**< 0.0001**
Turbidity	0.05	**< 0.0001**	0.03	**0.0006**	0.04	**< 0.0001**	0.02	**0.0020**	0.06	**< 0.0001**	0.08	**< 0.0001**
pH	0.03	**< 0.0001**			0.07	**< 0.0001**			0.02	**< 0.0001**	0.04	**< 0.0001**
Depth	0.01	**0.0066**							0.02	**< 0.0001**	0.01	**< 0.0001**
X1	0.09	**< 0.0001**	0.09	**< 0.0001**	0.03	**0.0005**	0.09	**< 0.0001**	0.08	**< 0.0001**	0.36	**< 0.0001**
X2	0.07	**< 0.0001**	0.05	**< 0.0001**	0.11	**< 0.0001**	0.05	**< 0.0001**	0.07	**< 0.0001**	0.09	**< 0.0001**

Supplementary Table 7 | Partitioning of morphological and genomic variation in eight populations of the nine-spined stickleback. The variation in morphology and genomics is partitioned in environmental (ENV) and spatial (SPACE) components. For morphology, analyses were conducted for all 14 traits combined as well as for each trait category separately (armour, body shape and gill morphology; see Supplementary Table 2). Here, the dependent variables are the individual size-corrected phenotypic traits (residuals of a linear regression of each trait on standard length or gill arch length). For genomics, analyses were conducted for the neutral loci as well as for the outlier loci. Here, the dependent variables are the first principal components (PC_1 to PC_n) of the neutral and outlier SNPs, where n is the number of principal components needed to include 50 % of the total allelic variation. Environmental dimensions include salinity, water depth, pH, turbidity and macroinvertebrate predators (see Supplementary Table 1). Spatial dimensions include X1 and X2, which represent the first and second dimension of a classical multidimensional scaling analysis on waterway distances. The unique and shared fractions explained by the respective variance components are represented by adjusted R^2 (R^2 adj), and by R^2 for each explanatory variable. Significant P-values are in bold. N: sample size. TV: total variation.

	**All traits**		**Armour**		**Body shape**		**Gill morphology**		**Neutral loci**		**Outlier loci**	
**Effect**	**R^2 adj**	***P***	**R^2 adj**	***P***	**R^2 adj**	***P***	**R^2 adj**	***P***	**R^2 adj**	***P***	**R^2 adj**	***P***
N	126		153		158		130		175		175	
TV	1750		608		785		645		145304		414	
ENV	0.10	**< 0.0001**	0.10	**< 0.0001**	0.04	**0.0022**	0.16	**< 0.0001**	0.12	**< 0.0001**	0.41	**< 0.0001**
SPACE	0.02	**0.0041**	0.01	0.0533	0.00	0.2335	0.05	**0.0006**	0.06	**< 0.0001**	0.20	**< 0.0001**
ENV + SPACE	0.10	**< 0.0001**	0.11	**< 0.0001**	0.05	**0.0010**	0.16	**< 0.0001**	0.15	**< 0.0001**	0.45	**< 0.0001**
ENV|SPACE	0.09	**< 0.0001**	0.10	**< 0.0001**	0.04	**0.0004**	0.10	**< 0.0001**	0.09	**< 0.0001**	0.25	**< 0.0001**
SPACE|ENV	0.00	0.4815	0.01	0.1525	0.01	0.0557	0.00	0.7674	0.03	**< 0.0001**	0.04	**< 0.0001**
Residuals	0.90		0.89		0.95		0.84		0.85		0.55	
**Variable**	**R^2**	***P***	**R^2**	***P***	**R^2**	***P***	**R^2**	***P***	**R^2**	***P***	**R^2**	***P***
Salinity	0.02	**0.0131**	0.02	**0.0444**			0.06	**0.0002**	0.05	**< 0.0001**	0.34	**0.0001**
Invertebrates	0.03	**< 0.0001**	0.06	**< 0.0001**					0.03	**< 0.0001**	0.02	**0.0011**
Turbidity	0.05	**< 0.0001**	0.03	**0.0020**	0.03	**0.0020**	0.08	**0.0001**	0.03	**< 0.0001**	0.04	**0.0001**
pH	0.02	**0.0017**	0.02	**0.0141**					0.02	**< 0.0001**	0.02	**0.0009**
Depth	0.02	**0.0197**					0.03	**0.0080**	0.02	**< 0.0001**	0.02	**0.0002**
X1	0.02	**0.0102**					0.04	**0.0024**	0.03	**< 0.0001**	0.15	**< 0.0001**
X2	0.01	**0.0451**	0.02	**0.0165**			0.03	**0.0189**	0.04	**< 0.0001**	0.06	**< 0.0001**

SUPPLEMENTARY METHODS

Study area and species

The coastal lowlands of Belgium and the Netherlands harbour a diversity of aquatic habitats such as ponds, ditches, small streams, estuaries and polder creeks [1-3]. The area contains diked brackish and freshwater habitats of Holocene origin with variable connectivity to adjacent estuaries and the open sea. Habitats are usually shallow (< 1.5 m) and the water current is slow to stagnant. Three-spined stickleback (*Gasterosteus aculeatus* L.; Gasterosteidae; Teleostei) and nine-spined stickleback (*Pungitius pungitius* L.; Gasterosteidae; Teleostei) dominate the fish community. Both fishes are widely distributed in the northern hemisphere [4]. While both species occur in marine and freshwater environments, the three-spined stickleback is more euryhaline than the nine-spined stickleback, which is scarcely found in full seawater [4]. Paleoclimate and phylogeography suggest a postglacial expansion by marine populations recolonizing freshwater habitat for the three-spined stickleback [5, 6]. For the nine-spined stickleback, phylogeographical data and ecological features suggest that divergence has mainly proceeded in freshwater [7]. Populations in Belgium and the Netherlands belong to the western European clade in case of the three-spined stickleback [6], and to the north-western European clade in case of the nine-spined stickleback [7]. While the exact colonisation history of both species in our study area is unknown, its close proximity to the coast implies that the distribution of both species has been influenced until recently by a shifting coastline after the last glacial, and by the construction of polders and drainage systems.

Field work

Field sampling was done in accordance to European directive 2010/63/EU and explicit permission of the Agency for Nature and Forests. We selected eight sites, including four brackish sites and four freshwater sites (Supplementary Table 1; Figure 1). The sites were visited four times (spring 2008, fall 2008, spring 2009 and summer 2009) in order to obtain habitat characteristics, estimates of population density, and specimens. Field work was performed as described in [8]. In short, we started with monitoring the water for temperature, pH, and conductivity (µS/cm; for analyses converted to salinity in psu) using a Hach field-monitoring unit (Hach, Loveland, Co, USA). Sites with consistently low salinities (i.e. equivalent to conductivities < 1000 µS/cm) were classified as freshwater sites (brackish water sites had higher as well as more variable salinity values owing to the irregular influx of seawater; Supplementary Table 1). An index for water turbidity was obtained with a Snell tube [9]. Water depth was determined as the median depth (cm) of five equidistant points along a stretch of 100 m of each waterbody. A single person (JAMR) then sampled three-spined and nine-spined stickleback by progressively dipnetting along the bank of the same stretch of 100 m with approximately one dip per meter, each time using the same hand net. This method enabled us to estimate the density of three-spined and nine-spined stickleback as the number of individuals per meter along the bank. Given that all sites are shallow (i.e., < 75 cm depth: Supplementary Table 1), vegetated and narrow (i.e., < 3 meter), any catch bias across sites and species was probably negligible. While fishing, the total density of macro-invertebrate predators of stickleback was determined by counting all backswimmers (*Notonecta glauca*), dragonfly larvae (*Anax* sp. and *Aeschna* sp.) and large diving beetles (*Dytiscus marginalis*). Sticklebacks were immediately anaesthetized and flash frozen in dry ice after capture. A minimum of 24 adult individuals per site and species, all obtained in the spring of 2009, were selected for subsequent morphological and genomic characterisation. Final sample sizes (i.e., excluding individuals with missing data or low read number) are listed in Supplementary Table 1.

Morphological characterisation

In the lab, the sticklebacks were thawed on ice, measured for body size (standard length (SL); ± 0.1 cm), and weighed (± 0.01 g). The left side of each specimen was photographed with a scale bar from a standard camera position for morphometric analysis. A piece of the caudal fin was sampled and stored in 100% ethanol for DNA analysis. Individuals were then stored on a 4% formalin solution. After 2 months, the formalin-preserved fish were rinsed with water for 72 h, bleached for 4 h (1% KOH bleach solution), and stained with alizarin red S to facilitate plate counts [10]. After staining, the number of lateral plates on the left side was determined. The presence of a keel, a small modification of the caudal lateral plates, was noted, but not included in the plate count. Subsequently, the length of the pelvic plate (PP), the left pelvic spine (PS) and the first dorsal spine (DS1) were measured using a digital calliper (± 0.01 mm). Body depth (BD), the diameter of the eye (EYE), dorsal fin length (DF), anal fin length (AF) and tail length (Tail) were measured digitally using the software TPS v.2.18 [11]. Finally, the gill cover was removed to dissect the left part of the gills. With the aid of a stereomicroscope, the number of large gill rakers (NLGR) on the frontal and distal part of the first gill arch was determined. The length of the first branchial arch (GA), as well as the length of its second (LGR2), third (LGR3) and fourth (LGR4) gill raker, were measured under the stereomicroscope.

DNA extraction and genotyping-by-sequencing

For both species, 192 individuals (i.e., 24 individuals of each site) were selected for genotyping-by-sequencing (GBS) [12]. Compared to other reduced representation libraries such as RAD sequencing [13], GBS is a simple, quick and highly reproducible method for preparing libraries for the Illumina sequencing platform. Genomic DNA was extracted from fin clips using the Nucleospin 96 Tissue DNA Extraction kit (Macherey-Nagel) according to the manufacturers protocol. Purified DNA from each individual was digested with a methylation sensitive restriction enzyme *ApeKI* (GCWGC), followed by the ligation of a common and unique barcode adaptor. After ligation, samples were pooled, purified and size-selected by a PCR reaction with Illumina primers. Finally, each library was purified again, quantified and sent for sequencing using paired-end 100-bp reads on the Illumina HiSeq 2000 at the Genomics Core Facility of the University of Leuven (http://gc.uzleuven.be).

SNP genotyping

Reference genome SNP discovery pipeline - Reference genome based SNP genotyping was performed using the TASSEL GBS pipeline (Version 3) [14]. SNP calling and genotyping were performed by setting the restriction enzyme (-e *ApeKI*), as well as the minimum number of tags required for output (-c 5). The TAGs file was mapped to the three-spined stickleback genome using the software BWA [15]. The resulting genotype file in VCF format was used for further filtering. Individuals which had less than 500,000 reads, including 17 three-spined sticklebacks as well as 17 nine-spined sticklebacks, were removed - leaving 175 out of 192 individuals of each species. Only variants covered in at least 90 % of the individuals with minimum allele frequency of 0.05 and heterozygosity less than 0.5 were used. Heterozygosity filtering was performed to remove potential paralogs. We genotyped 12,858 SNPs in the three-spined stickleback and 3,877 SNPs in the nine-spined stickleback. In the three-spined stickleback, further linkage disequilibrium (LD) based filtering was performed to remove redundant information. Briefly, LD between pairs of sites was measured as the standard disequilibrium coefficient (D') and squared allele-frequency (r^2) implemented in the TASSEL software. One representative SNP of the SNPs which were found to be linked (P-values ≤ 0.05; Fisher exact test) was kept for population genomic analysis. The filtering step retained 12,754 SNPs.

De novo *SNP discovery pipeline* - The sequencing data analysis was performed using the UNEAK GBS (v3.0.169) pipeline [16], which is part of the TASSEL software [17]. This SNP discovery and genotyping method does not require a reference genome and works on the principle of network analysis. The UNEAK pipeline is based on single end sequencing data. The SNP calling and genotyping were performed by setting restriction enzyme (-e *ApeKI*), minimum number of tags required for output (-c 5), error tolerance rate (-e 0.03), minimum/maximum minor allele frequencies (-mnMAF 0.05/-mxMAF 0.5), and minimum/maximum call rates (-mnC 0 -mxC 1). The resulting genotype file in Hapmap format was converted to VCF format for further filtering. As for the RG-based pipeline, individuals with reads less than 500,000 were removed, leading to 175 individuals out of 192 for each species. In addition, only variants covered in at least 90 % individuals with a minimum allele frequency of 0.05 and heterozygosity less than 0.5 were used. Heterozygosity filtering was performed to remove potential paralogs. We genotyped 4,760 and 10,090 polymorphic markers for the three-spined and nine-spined stickleback, respectively.

Annotation of tag sequences – Annotation of tag sequences (FASTA format) obtained from the above filtered variants was performed using the BLAST software using the three-spined stickleback genome. The result was filtered for an E-value of 0.001. For the three-spined stickleback, 86.6 % of both the reference-based FASTQ sequence reads as well as the *de novo*-based FASTQ tags could be mapped to the three-spined stickleback genome. For the nine-spined stickleback, 18.3 % of the reference-based FASTQ sequence reads and 23.3 % of the *de novo*-based FASTQ tags could be mapped to the three-spined stickleback genome. In comparison, previous studies found that 47.5 % of consensus sequences and 42 % of the transcriptome of the nine-spined stickleback mapped to the three-spined stickleback genome [18, 19].

Data analysis

Analyses aimed at comparing both species for phenotypic differentiation, SNP-based neutral genetic structure and SNP-based signatures of selection. Subsequently, a variance partitioning approach was used to estimate the contribution of spatial and environmental factors to phenotypic, neutral allelic variation and putatively adaptive allelic variation. All analyses (unless specified) were performed using the statistical software package R (www.r-project.org).

Phenotypic differentiation – MANOVAs and ANOVAs were used to examine how site and body size influence variation in single and combined phenotypic traits in each species separately, as well how site, body size and species influence variation in phenotypic traits across both species combined. Models across both species combined also included the species by body size interaction term, allowing us to account for separate slopes in both species. We also added the site by species interaction term to these models, such that both parallel (effect of site), species-specific (effect of species) and non-parallel (effect of site by species interaction) effects could be quantified. To do so, partial η^2 values (in the case of MANOVA) and R^2 values (in the case of ANOVA) of each of term in each model were calculated. The relative importance of the parallel vs. non-parallel contribution to the variation of each trait was then calculated as $R^2[par]/R^2[npar]$, where $R^2[par]$ and $R^2[npar]$ quantify the parallel and non-parallel effect size, respectively. Furthermore, to explicitly test which phenotypic traits differ between populations from freshwater and brackish water habitat, we also performed ANOVAs with site nested in habitat type. Finally, in order to compare the level of phenotypic differentiation directly with the level of genetic differentiation, we calculated P_{ST}, an index which quantifies the proportion of among population phenotypic variance in quantitative traits [20]. P_{ST} estimation was performed using a Bayesian approach following Leinonen *et al.* [21]. Specifically, traits were assumed to be normally distributed, and a linear model was fitted to each trait separately. Population was entered into the model as a random effect, and body size as a covariate. The models were fitted to the data using a Gibbs sampler implemented in the software WinBUGS 1.4 [22]. Prior distributions for each trait were uninformative, and posterior distributions were obtained by running five independent chains (50 000 iterations) after a burn-in of 1000 iterations.

Neutral genetic structure – First, genetic diversity in each population and species was calculated as the expected heterozygosity (H_E) as implemented in the HIERFSTAT package in R. This metric was then used to test whether or not genetic diversity declines with Euclidian distance to the coast in each species. Second, effective population size (N_e) was estimated using the software NeEstimator (version 2.01) [23]. N_e was calculated using the linkage-disequilibrium method, assuming random mating and excluding alleles with frequencies < 0.05. Third, overall and pairwise population differentiation was quantified with the ADEGENET and DIVERSITY package in R using the standardized allelic variance F_{ST} [24]. Pairwise F_{ST} values were used to visualize

population structure with a two-dimensional classical multidimensional scaling (CMDS) plot with the function cmdscale in R. Finally, we also assessed population structure in both species using a Bayesian framework implemented in fastSTRUCTURE [25]. The most likely population structure was chosen based on the maximal marginal likelihood according to a specific number of groups ($1 \leq K \leq 8$).

Genomic signatures of selection – Three methods were used to conduct global outlier tests across populations [26]. First, we used LOSITAN, which implements an island model and uses a coalescent method to determine the distribution of F_{ST} as a function of the heterozygosity [27]. We ran the function that first establishes a neutral F_{ST} baseline by removing putative markers under selection, defined as markers that fall outside the 95% interval. We ran 10^5 simulations as recommended [27]. Second, we performed the outlier detection method implemented in ARLEQUIN v3.5.2.3 [28]. This method implements the same island model as LOSITAN, but adds on the option for hierarchical clustering. In the presence of strong hierarchical population structure it reduces false positives by a hierarchical analysis of genetic differentiation [29]. To do so, we clustered populations according to the population structure suggested by fastSTRUCTURE (see above). We used the standard settings of 20,000 simulations for each run and 100 demes per group. Finally, we used BAYESCAN v2.01 [30] running a logistic regression model which explains the observed genetic diversity by dividing it in a locus- and a population-specific component [31]. The method allows for different migration rates and population sizes and thus can be used for scenarios that deviate from the island model [32]. We set the prior odds of neutrality to 100. We conducted 10 pilot runs of 5,000 iterations, followed by an additional 150,000 iterations and a burn-in of 50,000 iterations.

In order to control the false discovery rate (FDR), as well as to assess statistical significance in a comparable way across methods [33], P-values (in the case of LOSITAN and ARLEQUIN) and posterior probabilities (in the case of BAYESCAN) were transformed into q-values, and statistical significance of these values was evaluated at a q-value cut-off of 0.05. In order to further reduce the number of false positives, all methods were executed three times, i.e. only the loci that were thrice significant ($q < 0.05$) were appointed as outliers. We detected outlier loci that are under balancing and directional selection. However, any tests of balancing selection based on F_{ST} are inaccurate [26, 34], and therefore we only evaluated the loci under directional selection. The number as well as the percentage of such loci were calculated and compared among the two species, and this was done for each method separately. Neutral and outlier loci were then split into separate matrices in order to further analyse and compare patterns of neutral and non-neutral population divergence. Also this step was done for each method separately, in particular because the overlap in outliers between methods was relatively small, especially between BAYESCAN on the one hand and ARLEQUIN and LOSITAN on the other hand. Yet, patterns of neutral and non-neutral divergence were quantitatively and qualitatively very similar across methods. We therefore only present the results obtained with BAYESCAN, which was the method which resulted in the smallest (i.e. most conservative) difference in the proportion of outliers between both species.

As an alternative approach to assess the adaptive significance of the SNPs, we performed an association analysis between the environmental variables and all loci using a latent factor mixed model (LFMM) approach [35]. LFMM uses MCMC simulations while controlling for population structure using latent factors. The number of latent factors was set equal to K, the number of clusters as determined by fastSTRUCTURE (see above). The correlation between a SNP and an environmental variable was deemed significant at the 5 % level. We then compared both species for the percentage of outlier loci (as identified with BAYESCAN) that were correlated with each of the environmental variables.

Variance partitioning – For each species, we conducted a redundancy analyses (RDA) [36] to partition the explainable phenotypic variation, neutral allelic variation and allelic variation at outlier loci (as identified with BAYESCAN) into those attributable to spatial factors (SPACE; indicative of spatial isolation), environmental factors (ENV; indicative of the response to divergent selection), and their joint effect (indicative of congruent effects of spatial isolation and divergent selection). We first tested the null hypothesis that each set of explanatory variables separately (SPACE or ENV) does not explain phenotypic or genetic variation. Subsequently, we quantified and tested the partial and combined contribution of SPACE and ENV to the explainable phenotypic or genetic variation. Variance components were estimated and tested for significance using 10^3 random permutations of the data. Analyses were performed using the VEGAN package in R [37]. In the cases where the RDA analysis was significant, we applied forward selection in order to determine the most influential single explanatory variables. For this purpose, we used the procedure implemented in the PACKFOR package in R [38].

Genome-wide visualisation and gene ontology - For both species, Circos plots [39] were constructed to visualise the genotyped loci and their F_{ST} distribution throughout the three-spined stickleback genome [40]. Outlier loci (as identified with BAYESCAN) were mapped on the three-spined stickleback genome and were used as a reference to determine the genes 5 kb upstream and downstream from these loci. The identified genes were then used to map gene ontology (GO) terms using the PANTHER database with *Homo sapiens* as option [41].

SUPPLEMENTARY REFERENCES

1. Raeymaekers JAM, Maes GE, Audenaert E, Volckaert FAM. Detecting Holocene divergence in the anadromous-freshwater three-spined stickleback (*Gasterosteus aculeatus*) system. *Mol Ecol* **14**, 1001-1014 (2005).
2. Van Dongen S, Lens L, Pape E, Volckaert FAM, Raeymaekers JAM. Evolutionary history shapes the association between developmental instability and population-level genetic variation in three-spined sticklebacks. *J Evol Biol* **22**, 1695-1707 (2009).
3. Raeymaekers JAM, Huyse T, Maelfait H, Hellemans B, Volckaert FAM. Community structure, population structure and topographical specialisation of *Gyrodactylus* (Monogenea) ectoparasites living on sympatric stickleback species. *Folia Parasitol* **55**, 187-196 (2008).
4. Wootton RJ. *The Biology of the Sticklebacks.* Academic Press (1976).
5. Bell MA, Foster SA. *The Evolutionary Biology of the Threespine Stickleback. Oxford Science Publications, Oxford University Press* (1994).
6. Mäkinen HS, Merilä J. Mitochondrial DNA phylogeography of the three-spined stickleback (*Gasterosteus aculeatus*) in Europe - Evidence for multiple glacial refugia. *Mol Phylogenet Evol* **46**, 167-182 (2008).
7. Wang C, Shikano T, Persat H, Merilä J. Mitochondrial phylogeography and cryptic divergence in the stickleback genus *Pungitius*. *J Biogeogr* **42**, 2334-2348 (2015).
8. Raeymaekers JAM, Konijnendijk N, Larmuseau MHD, Hellemans B, De Meester L, Volckaert FAM. A gene with major phenotypic effects as a target for selection vs. homogenizing gene flow. *Mol Ecol* **23**, 162-181 (2014).
9. Louette G, De Meester L. High Dispersal Capacity of Cladoceran Zooplankton in Newly Founded Communities. *Ecology* **86**, 353-359 (2005).
10. Taylor WR, Dyke GCV. Revised procedures for staining and clearing small fishes and other vertebrates for bone and cartilage study. *Cybium* **9**, 107-119 (1985).
11. Rohlf FJ. tpsDig, Digitize Landmarks and Outlines. Department of Ecology and Evolution, State University of New York at Stony Brook (2012).
12. Elshire RJ*, et al.* A robust, simple genotyping-by-sequencing (GBS) approach for high diversity species. *PLoS One* **6**, (2011).
13. Hohenlohe PA, Bassham S, Etter PD, Stiffler N, Johnson EA, Cresko WA. Population genomics of parallel adaptation in threespine stickleback using sequenced RAD tags. *PLoS Genet* **6**, 23 (2010).
14. Glaubitz JC*, et al.* TASSEL-GBS: a high capacity genotyping by sequencing analysis pipeline. *PLoS One* **9**, 11 (2014).
15. Li H, Durbin R. Fast and accurate short read alignment with Burrows-Wheeler transform. *Bioinformatics* **25**, 1754-1760 (2009).
16. Lu F*, et al.* Switchgrass genomic diversity, ploidy, and evolution: novel insights from a network-based SNP discovery protocol. *PLoS Genet* **9** (2013).
17. Bradbury PJ, Zhang Z, Kroon DE, Casstevens TM, Ramdoss Y, Buckler ES. TASSEL: software for association mapping of complex traits in diverse samples. *Bioinformatics* **23**, 2633-2635 (2007).
18. Bruneaux M, Johnston SE, Herczeg G, Merilä J, Primmer CR, Vasemagi A. Molecular evolutionary and population genomic analysis of the nine-spined stickleback using a modified restriction-site-associated DNA tag approach. *Mol Ecol* **22**, 565-582 (2013).
19. Guo BC, Chain FJJ, Bornberg-Bauer E, Leder EH, Merilä J. Genomic divergence between nine- and three-spined sticklebacks. *BMC Genomics* **14** (2013).
20. Raeymaekers JAM, Van Houdt JKJ, Larmuseau MHD, Geldof S, Volckaert FAM. Divergent selection as revealed by P_{ST} and QTL-based F_{ST} in three-spined stickleback (*Gasterosteus aculeatus*) populations along a coastal-inland gradient. *Mol Ecol* **16**, 891-905 (2007).
21. Leinonen T, Cano JM, Mäkinen H, Merilä J. Contrasting patterns of body shape and neutral genetic divergence in marine and lake populations of threespine sticklebacks. *J Evol Biol* **19**, 1803-1812 (2006).
22. Spiegelhalter DJ, Thomas A, Best NG, Lunn D. *WinBUGS. Version 1.4. User manual* (2003).
23. Do C, Waples RS, Peel D, Macbeth GM, Tillett BJ, Ovenden JR. NEESTIMATOR v2: re-implementation of software for the estimation of contemporary effective population size (N_e) from genetic data. *Mol Ecol Resour* **14**, 209-214 (2014).
24. Jost L. G_{ST} and its relatives do not measure differentiation. *Mol Ecol* **17**, 4015-4026 (2008).
25. Raj A, Stephens M, Pritchard JK. fastSTRUCTURE: variational inference of population structure in large SNP data sets. *Genetics* **197**, 573-U207 (2014).
26. Narum SR, Hess JE. Comparison of F_{ST} outlier tests for SNP loci under selection. *Mol Ecol Resour* **11**, 184-194 (2011).
27. Antao T, Lopes A, Lopes RJ, Beja-Pereira A, Luikart G. LOSITAN: a workbench to detect molecular adaptation based on a F_{ST}-outlier method. *BMC Bioinformatics* **9** (2008).
28. Excoffier L, Lischer HE. Arlequin suite ver 3.5: a new series of programs to perform population genetics analyses under Linux and Windows. *Mol Ecol Resour* **10**, 564-567 (2010).
29. Excoffier L, Hofer T, Foll M. Detecting loci under selection in a hierarchically structured population. *Heredity* **103**, 285-298 (2009).
30. Foll M, Gaggiotti O. A genome-scan method to identify selected loci appropriate for both dominant and codominant markers: a Bayesian perspective. *Genetics* **180**, 977-993 (2008).
31. Beaumont MA, Balding DJ. Identifying adaptive genetic divergence among populations from genome scans. *Mol Ecol* **13**, 969-980 (2004).
32. Whitlock MC, Lotterhos KE. Reliable detection of loci responsible for local adaptation: inference of a null nodel through trimming the distribution of F_{ST}. *The American Naturalist* **186**, S24-S36 (2015).

33. Lotterhos KE, Whitlock MC. Evaluation of demographic history and neutral parameterization on the performance of F_{ST} outlier tests. *Mol Ecol* **23**, 2178-2192 (2014).
34. Hansen MM, Meier K, Mensberg K-LD. Identifying footprints of selection in stocked brown trout populations: a spatio-temporal approach. *Mol Ecol* **19**, 1787-1800 (2010).
35. Frichot E, Schoville SD, Bouchard G, Francois O. Testing for associations between loci and environmental gradients using latent factor mixed models. *Mol Biol Evol* **30**, 1687-1699 (2013).
36. Borcard D, Gillet F, Legendre P. *Numerical ecology with R*. Springer (2011).
37. Oksanen J*, et al.* vegan: Community Ecology Package. R package version 2.3-1 (2015).
38. Dray S, Legendre P, Blanchet G. packfor: Forward Selection with permutation (Canoco p.46). R package version 0.0-8/r109 (2013).
39. Krzywinski M*, et al.* Circos: an information aesthetic for comparative genomics. *Genome Res* **19**, 1639-1645 (2009).
40. Jones FC*, et al.* The genomic basis of adaptive evolution in threespine sticklebacks. *Nature* **484**, 55-61 (2012).
41. Mi H*, et al.* PANTHER version 11: expanded annotation data from gene ontology and reactome pathways, and data analysis tool enhancements. *Nucleic Acids Res* **45**, D183-D189 (2017).
